# Immunomodulatory Role of the Antimicrobial LL-37 Peptide in Autoimmune Diseases and Viral Infections

**DOI:** 10.3390/vaccines8030517

**Published:** 2020-09-10

**Authors:** Bapi Pahar, Stefania Madonna, Arpita Das, Cristina Albanesi, Giampiero Girolomoni

**Affiliations:** 1 Division of Comparative Pathology, Tulane National Primate Research Center, Covington, LA 70433, USA; 2 Department of Microbiology and Immunology, Tulane University School of Medicine, New Orleans, LA 70118, USA; 3 IDI-IRCCS, Dermopathic Institute of the Immaculate IDI, 00167 Rome, Italy; s.madonna@idi.it (S.M.); c.albanesi@idi.it (C.A.); 4 Division of Microbiology, Tulane National Primate Research Center, Covington, LA 70433, USA; arpita@tulane.edu; 5 Section of Dermatology, Department of Medicine, University of Verona, 37126 Verona, Italy; giampiero.girolomoni@univr.it

**Keywords:** cathelicidin, LL-37, lupus erythematosus, psoriasis, virus

## Abstract

Antimicrobial peptides (AMPs) are produced by neutrophils, monocytes, and macrophages, as well as epithelial cells, and are an essential component of innate immunity system against infection, including several viral infections. AMPs, in particular the cathelicidin LL-37, also exert numerous immunomodulatory activities by inducing cytokine production and attracting and regulating the activity of immune cells. AMPs are scarcely expressed in normal skin, but their expression increases when skin is injured by external factors, such as trauma, inflammation, or infection. LL-37 complexed to self-DNA acts as autoantigen in psoriasis and lupus erythematosus (LE), where it also induces production of interferon by plasmocytoid dendritic cells and thus initiates a cascade of autocrine and paracrine processes, leading to a disease state. In these disorders, epidermal keratinocytes express high amounts of AMPs, which can lead to uncontrolled inflammation. Similarly, LL-37 had several favorable and unfavorable roles in virus replication and disease pathogenesis. Targeting the antiviral and immunomodulatory functions of LL-37 opens a new approach to limit virus dissemination and the progression of disease.

## 1. Introduction

The innate immune system plays a crucial role in protection against microbes, as well as in the initiation of inflammatory responses. Antimicrobial peptides (AMPs) constitute an important component of innate immunity against bacteria, fungi, protozoal, and viral infections [1,2,3]. In addition, AMPs exert numerous immunomodulatory properties by inducing cytokine production, chemoattraction, and immune cell differentiation, thus linking innate to adaptive immunity [4,5,6,7]. Three major AMP classes are the cathelicidin, β-defensins, and S100 proteins. Cathelicidins are a family of α-helical peptides containing LL-37 [8,9]; β-defensins are a family of β-sheet peptides containing human beta-defensin (hBD)1, hBD2 [10], and hBD3 [11]; and S100 proteins contain S100A7 (psoriasin), which is abundantly expressed in psoriatic skin [12,13]. Similarly α-, β-, θ-defensins, including HNP1-3, human defensins (HD)5, and cathelicidins, have been shown to have antiviral effects against several viral diseases, including herpes simplex virus (HSV) types 1 and 2, human immunodeficiency virus (HIV), cytomegalovirus (CMV), and vesicular stomatitis viruses [2,14,15]. AMPs can be produced by a variety of cell types, including keratinocytes, neutrophils, monocytes, and macrophages. AMPs are scarcely expressed in normal skin, but their expression increases when skin is injured by external factors, such as trauma, inflammation, or infection [16]. The aberrant expression of AMPs can lead to uncontrolled inflammation in autoimmune diseases, like psoriasis and lupus erythematosus (LE) [17,18,19,20,21,22], and may accelerate viral infections [23]. 

LL-37, the only human member of cathelicidin family, is an amphipathic, positively-charged 37-residue peptide generated from the precursor hCAP18 protein, which is stored in the secondary granules of neutrophils, from where it is released upon activation [24]. LL-37 has a secondary alpha helix structure and amphipathic properties that allow its interaction with bacterial membranes or other anionic components [24]. The hydrophobic portion contains positively charged residues that interact with negatively charged molecules, such as lipopolysaccharides (LPSs), DNA/RNA molecules, and the bacterial cell wall. Its cationic, amphipathic alpha helix structure has three domains: a N-terminal alpha helix, a C-terminal alpha helix, and a C-terminal tail. The N-terminal alpha helix is involved in the chemotaxis of innate immune cells and has hemolytic activity in humans. The C-terminal alpha helix is responsible for antimicrobial and antiviral activity of LL-37. Finally, the C-terminal tail contributes to the formation of peptide tetramers, interacting primarily with negatively charged molecules, such as anionic phosphatidylglycerols, LPSs of Gram-negative bacteria, and teichoic acid of Gram-positive bacteria [24]. This domain is responsible for target specificity against bacterial anionic membranes, while protecting eukaryotic cationic membranes, which are instead composed of cholesterol and phospholipids. In human keratinocytes, the expression of LL-37 and other AMPs can be regulated by various exogenous (bacterial and viral stimuli) and endogenous factors, such as pro-inflammatory cytokines (i.e., interleukin (IL)-17A, IL-36γ), growth factors, and the active form of vitamin D [25,26,27]. Recently, van Harten et al. summarized the structure and biological functions of cathelicidins, including the human LL-37, focusing on their pro-inflammatory and anti-inflammatory properties, as well on their direct and indirect effects on chemotaxis and cell differentiation [28]. Additionally, the authors discussed the potential and limitations of using cathelicidins as immunomodulatory (including vaccine adjuvants) or antimicrobial drugs. 

In this review, we will dissect the role of the human cathelicidin LL-37 in the dysregulation of innate immune pathways occurring in autoimmune diseases, including psoriasis and LE, as well as in some viral diseases, with a particular focus on its immunomodulatory functions and its potential as a novel therapeutic approach.

## 2. LL-37 in Autoimmune Diseases

Psoriasis is a chronic inflammatory skin disorder resulting from genetic and environmental factors, in which disturbances of innate and adaptive cutaneous immune responses lead to uncontrolled keratinocyte proliferation and dysfunctional differentiation [29]. In psoriasis, IL-17A-producing CD4^+^ and CD8^+^ T cells play a key pathogenic role. These T cells emerge following the local exposure and presentation of autoantigen(s) by tumor necrosis factor (TNF)-α- and IL-23-releasing dendritic cells [30,31]. To date, at least four autoantigens have been identified in psoriasis, including LL-37, keratin 17, and the disintegrin and metalloprotease domain containing thrombospondin type 1 motif-like 5 (ADAMTSL5), as well as neolipids generated by mast cell phospholipase A2 group IVD and presented by CD1a^+^ dendritic cells [21,32,33,34].

**Figure 1:**
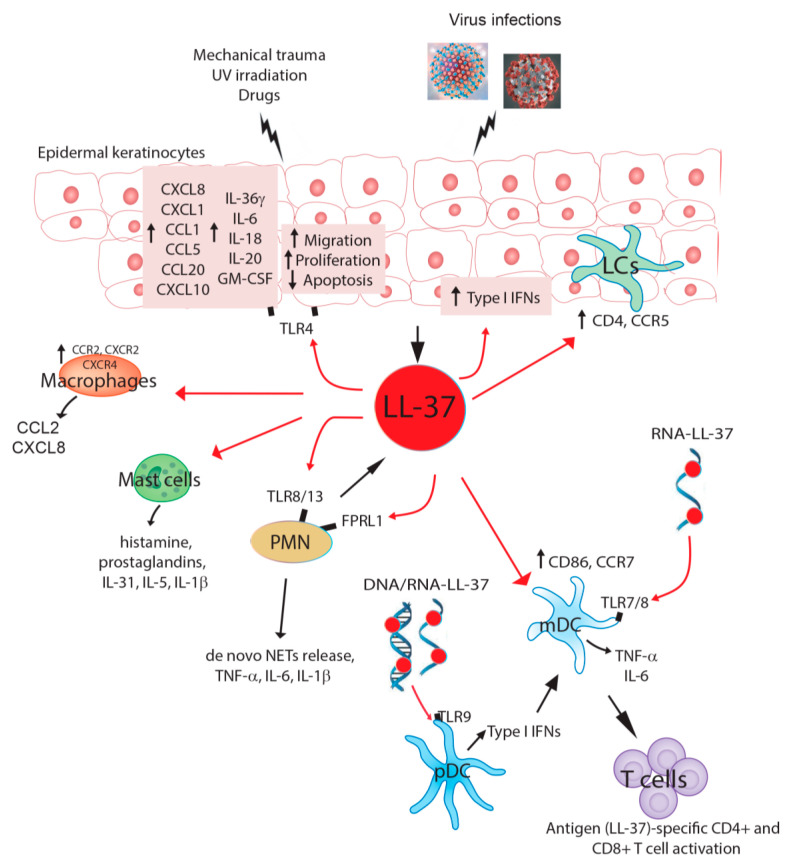
Graphical review of the main cellular targets of cathelicidin LL-37 in pathological skin conditions and viral infection. Mechanical trauma, UV irradiation, drugs, and viral infections can trigger LL-37 release by epidermal keratinocytes. In an autocrine loop, LL-37 induces the release of inflammatory cytokines, chemokines, and growth factors by keratinocytes themselves, which contribute to the skin recruitment and activation of innate immunity cells, including macrophages, mast cells, and polymorphonuclear neutrophils (PMN). LL-37 also impairs the apoptosis of epidermal keratinocytes, whereas it stimulates their proliferation and migration. LL-37 has direct effects also on innate immunity cells, inducing the release of pro-inflammatory mediators by macrophages (i.e., C-C Motif Chemokine Ligand (CCL)2 and C-X-C motif chemokine ligand (CXCL)8) and mast cells (i.e., histamines, prostaglandins, and IL-1b). Polymorphonuclear neutrophils (PMNs) are another source of LL-37, which may autocrinally trigger cytokine and *de novo* neutrophil extracellular trap (NET) release by naïve PMNs via Toll-like receptor (TLR) 8/13 and formyl peptide receptor-like 1 (FPRL1). In skin inflammation related to psoriasis, LL-37 complexed to DNA or RNA can also directly act on adaptive immunity cells, such as plasmacytoid dendritic cells (pDC), inducing the release of type I IFNs via TLR9, as well as on myeloid DC (mDC), inducing TNF-α and IL-6 production via TLR7/8. These events determine the expansion and activation of LL-37-specific CD4+/CD8+ T cell responses, with the development of skin psoriatic lesions. In skin injured by viral infections, LL-37 upregulates CD4 and CCR5 cell surface expression in Langerhans cells (LCs), thus increasing HIV susceptibility. In contrast, LL-37 upregulates CD86 and CCR7 expression in mDCs, decreasing HIV infection and transmission.

In the early events of psoriasis development, keratinocytes overproduce several innate immunity mediators, including IL-1 cytokines, chemokines, and AMPs—in particular LL-37, HBD-2, and psoriasin. These are involved in the activation and skin recruitment of innate immunity cells, such as plasmacytoid dendritic cells (pDC), neutrophils, and macrophages and mast cells (Figure 1) [27,29]. In 2007, Lande et al. demonstrated that LL-37 is the principal trigger of the pathogenic innate immune responses in psoriatic skin, providing the first link between an antimicrobial defense system and the pathogenesis of psoriasis [19]. In fact, LL-37 was found to be able to convert non-stimulatory self-DNA into a potent trigger of pDCs to produce interferon (IFN)-α, and thus to initiate innate and adaptive immune responses (Figure 1). This process involves first the binding of LL-37 to endogenous DNA through electrostatic interactions, and then the translocation of LL-37-DNA complexes into the endocytic pathway of pDCs, thereby bypassing a safety mechanism for discriminating viral/microbial from self-nucleic acids provided by the intracellular localization of Toll-like receptor (TLR) 9 [35]. Finally, LL-37 retains the DNA complex in early endocytic organelles, leading to IFN-α production by pDCs. TNF-α released by an activated pDC favors the maturation of myeloid dendritic cells, which in turn initiate cutaneous, lymphocyte-mediated autoimmune reactions. In this way, LL-37 released during skin injury breaks innate tolerance to self-DNA, and in a similar way to viral responses, elicits innate and adaptive immune responses, leading to the development of skin lesions [19]. Self-RNA–LL-37 complexes also trigger the activation of classical myeloid DCs (mDCs). This occurs through TLR7 and TLR8, and leads to the production of TNF-α and IL-6, as well as the differentiation of mDCs into mature DCs (Figure 1) [36]. However, LL-37 is not the only factor exerting this activity. Indeed, hBD2, hBD3, and lysozyme also activate pDCs by promoting self-DNA-mediated activation of endosomal TLR9, suggesting that cationic AMPs may have evolved from being pure antibiotics into promoters of host cell death sensing and initiators of immune responses [21] (Table 1).

Of note, both CD4^+^ and CD8^+^ LL-37-reactive T cells have been identified in patients with moderate-to-severe plaque psoriasis [22]. In fact, LL-37 peptides can determine the activation of CD4^+^ T cells by binding to the Human leukocyte antigen (HLA)-DR of dendritic cells, as well as that of CD8^+^ T cells, through their exposure to Major Histocompatibility Complex (MHC)class I complexes. While both CD4^+^ and CD8^+^ LL-37-specific T cells produce IFN-γ, only the CD4^+^ T-cell subtype also produces IL-17 cytokines [22]. The numbers of circulating LL-37-specific T cells significantly correlate with disease activity, suggesting their active contribution to disease pathogenesis [22]. 

Epidermal keratinocytes are the “first-line” skin cells responding to injury, and they may act as the earlier source of LL-37, HBD-2, and HBD-3. They can release LL-37 upon UV irradiation or physical trauma, as well as stimulation with bacterial or viral products (i.e., flagellin, LPSs, or viral RNA/DNA) or innate primary cytokines, such as IL-36γ, thus triggering paracrine and autocrine inflammatory loops [27,29,37,38]. Keratinocyte-derived LL-37 induces the release of IL-1 cytokines, including IL-36γ, by psoriatic keratinocytes themselves (Figure 1) [39].

Another source of nucleic acid-LL-37 complexes are neutrophils. In fact, following an initial activation of neutrophils by RNA from damaged keratinocytes, RNA complexed with LL-37 is abundant in neutrophil extracellular traps (NETs), and may trigger TLR8/TLR13-mediated cytokine and *de novo* NET release by naїve human neutrophils. In this way, neutrophil-mediated release of DNA/RNA and LL-37 complexes would enable pDCs and other immune cells to join the vicious cycle of self-propagating inflammation fueled by endogenous nucleic acids [40]. These findings raise the intriguing possibility that not only self-RNA, but also foreign, e.g., bacterial RNA or possibly fungal RNA, may exert immunostimulatory functions in the presence of LL-37 triggered during minor skin injury. However, further investigations need to substantiate this hypothesis (Table 1, Figure 1).

As mentioned above, other than affecting immune cells, LL-37 influences keratinocyte functions. Indeed, human cathelicidin induces migration and proliferation [41,42], and suppresses apoptosis of epidermal keratinocytes [43]. LL-37 by itself stimulates keratinocytes to synthesize and release different pro-inflammatory and immunoregulatory cytokines, including IL-6, IL-18, IL-20, Granulocyte-macrophage colony-stimulating factor (GM-CSF) [41,44,45], which are important for the recruitment and activation of neutrophils, but also the anti-inflammatory IL-10 cytokine. Furthermore, via IL-36R signaling, LL-37 induces CXCL8 and CXCL1 chemokines, which in turn recruit and induce a burst of neutrophils in lesional skin, typical of the early phase of psoriasis [39]. In addition, LL-37 induces CCL2, CCL5, CXCL10, and CCL20 chemokines in psoriatic keratinocytes, which contribute to recruitment of neutrophils and Th1/17 lymphocytes (Figure 1) [39,41,44,45]. 

Up to 30% of psoriasis patients develop psoriatic arthritis (PsA), a type of spondyloarthritis characterized by enthesitis, dactylitis, peripheral arthritis, and axial involvement, and skin lesions of psoriasis often precede PsA by 5–10 years [1]. Fresca et al. recently demonstrated that LL-37 is highly upregulated in the synovial fluids of PsA patients, and it becomes the target of autoantibodies, representing a novel autoantigen in PsA [46]. The presence of autoantibodies to native LL-37 in synovial fluids of PsA patients correlates well with several inflammatory markers and disease activity [46]. In addition, in this study, novel players/mechanisms, including LL-37 and its post-translational modifications (carbamylation and citrullination) have been found to be pathogenic in PsA. In particular, the authors reported that LL-37 is released by degranulating/netting neutrophils under the effect of inflammatory factors, such as GM-CSF and the complement component C5a, in PsA synovia, and it behaves as a novel B-cell autoantigen [46]. The formation of IgG-immune complexes (IgG-IC), including anti-LL-37 antibodies (Abs), and their deposition in synovial tissues of PsA can fuel this vicious inflammatory circle, and also induce a type-I signature via TLR7/8/9, as observed in systemic LE (SLE) [20]. Apart from psoriasis, other diseases are characterized by aberrant LL-37 expression. They include LE and rheumatoid arthritis (RA), where LL-37 is present in affected organs, such as the skin and kidneys, or in synovial fluids and circulation, respectively [47,48,49] (Table 1).

LE is an autoimmune disease with diverse and complicated etiology, including SLE and cutaneous LE (CLE), caused by the activation of autoreactive B cells producing autoantibodies against self-nucleic acids and associated proteins, such as LL-37 [50]. These Abs bind self-nucleic acids released by dying cells and form immune complexes that are deposited in different parts of the body, leading to detrimental inflammation and tissue damage [51,52]. CLE usually presents as one of manifestations of SLE patients; however there are also a proportion of SLE patients present without cutaneous manifestations [53]. The skin is the primarily affected organ in CLE, where it is peculiarly photosensitive to UV light, which can induce new skin lesions and exacerbate existing CLE disease.

Anti-microbial peptide LL-37 has been found accumulated also in lesional skin of CLE [54]. Similarly to lesional skin of psoriasis, anionic self-DNA is found complexed to LL-37, and these complexes are able to trigger TLR9-mediated type I IFNs production in pDCs in CLE [55]. Moreover, LL-37 contributes to DNA-mediated activation of CLE-derived keratinocytes, by inducing IFN-α expression [56].

In SLE, LL-37 is involved in an intriguing link between neutrophils, pDC activation, and autoimmunity. LL-37/self-DNA complexes are abundantly released by neutrophils in the form of NETs in SLE and they efficiently activate pDC via TLR9 [20]. In addition, similarly to PsA, SLE patients were found to develop autoAbs to both the self-DNA and LL-37 in NETs, indicating that these complexes could also serve as autoantigens triggering B-cell activation [20]. Circulating neutrophils from SLE patients release more NETs than those from healthy donors, and this is further stimulated by the AMP autoAbs, suggesting a mechanism for the chronic release of immunogenic complexes in SLE [20]. Finally, the high levels of type-I IFNs induce an unabated differentiation of monocytes into dendritic cells that stimulate autoreactive B cells and T cells [57], and decrease the threshold activation of autoreactive B cells [58], thereby promoting autoimmunity in SLE. Recently, Gestermann et al. demonstrated that neutrophils undergoing NET-induced cell death (named NETosis) expose their DNA complexed to LL-37 in SLE [59]. These structures not only triggered polyclonal B-cell activation through TLR9, but also stimulated NET-specific, self-reactive B cells by simultaneously engaging the B-cell receptor. In fact, via this mechanism, the increased NET formation in SLE patients triggered the activation of NET-specific, self-reactive B cells that produced pathogenic anti-LL-37 Abs, and potentially, anti-DNA Abs [59]. These findings suggest a link between neutrophils and B cells, in which NETs trigger a concerted activation of TLR9 and B-cell receptors, leading to anti-NET autoAbs production in SLE.

In addition to anti-LL-37 antibodies, 45% of SLE patients have circulating LL-37-specific T cells, which correlate with anti-LL37 Abs/disease activity [60]. However, in contrast to psoriatic Th17-cells, these LL-37-specific SLE T cells display a T-follicular helper (T_FH_)-like phenotype, with CXCR5/Bcl-6 and IL-21 expression, implicating their role in the stimulation of pathogenic Abs. Accordingly, LL-37-specific T-cells promoted B-cell secretion of pathogenic anti-LL-37 Ab production in vitro [60]. Therefore, in SLE, LL-37-specific T cells have a distinct functional specialization and antigenic specificity, suggesting that autoantigenic specificity is independent of the nature of the autoantigen, but rather relies on the disease-specific milieu driving T-cell subset polarization and autoantigen modifications.

Elevated expression of LL-37 and its activating protease have been described also in RA patients [47,61], even though mechanistic studies are necessary to unveil the role of LL-37 in this disease. Periarticular osteopenia is a common finding among patients with RA [62], and LL-37 induces the apoptosis of osteoblasts, which could contribute to reduced bone formation in arthritic joints [63]. Moreover, it has been demonstrated that in RA patients, neutrophils are prone to NETosis, and NETs are a source of citrullinated antigens and LL-37 in response to infections and toxins [64,65]. NETs may promote aberrant adaptive and innate immune responses in the joint and in the periphery, and perpetuate pathogenic mechanisms in this disease [66]. Finally, in RA patients, LL-37 supports the complement C1q in increasing NET-stimulatory activity on macrophages, due to the higher expression of C1q receptors, thus contributing to the inflammatory circuits occurring in RA [67].

## 3. Effect of LL-37 on DNA Viruses

### 3.1. Vaccinia Virus (VV)

Smallpox has been eradicated since 9 December 1979, but it has still been considered as a select agent, due to its potential use as a bioterrorism agent. Vaccinia virus (VV), a large, complex, and enveloped virus that belongs to *poxviridae* family, was used as a vaccine for the successful eradication of smallpox. LL-37 and not α or β defensins possess antiviral activity against VV [68]. The replication of VV in human keratinocytes in an in vitro cell culture system can be significantly inhibited by adding as low as 25 μM of LL-37 at 6 h after VV infection (Table 2). VV induces LL-37 production through the TLR3 pathway, and the presence of IL-4 and IL-13 inhibits the induction of LL-37 in VV-infected keratinocytes [69]. Cathelin-related antimicrobial peptide (CRAMP)-deficient mice had a higher level of VV replication compared to the wild type mice, which suggests that cathelicidins are the key regulator in controlling VV infection [68]. Increased VV replication in AD (atopic dermatitis) skin is correlated with decreased LL-37 upregulation, suggesting that LL-37 has a limited role in regulating VV replication in AD patients [69]. Therefore, LL-37 can be considered as a potential therapeutic approach for the initial control of VV replication and immunoregulation.

### 3.2. Herpes Simplex Virus 1 and 2 (HSV-1 and 2)

Neurotropic herpes simplex virus 1 (HSV-1) and type 2 (HSV-2), members of the human *Herpesviridae* family, are common human pathogens afflicting the oral and genital mucosa. Genital herpes poses a risk of 10 per 100,000 live births that infants will develop neonatal herpes during delivery, with an estimated 60% fatality without treatment [70,71]. In the United States from 1989–2010, 53% of pregnant women were seropositive for HSV-1, 9% were positive for HSV-2, and 15% were seropositive for both HSV-1 and HSV-2 [72]. Even today, no commercial vaccine is available to prevent HSV-1 and 2. LL-37 has been shown to have significant impact on HSV replication. A significant reduction in HSV-1 DNA titers was detected in human primary keratinocytes treated for 1 h with LL-37 compared to untreated cells. Keratinocytes infected with HSV-1 in the presence of LL-37 showed increased expression of five important interferon (IFN)-stimulated genes (ISGs) (IFIT1, OAS1, ISG20, IRF7, and viperin), and reduced the expression of IFN-β, suggesting that ISGs are key regulators to suppress HSV-1 replication in LL-37-treated in vitro cell cultures [73]. A significant reduction in HSV-1 replication has also been detected in in vitro cell culture assays where vero cells were pretreated with LL-37, followed by HSV-1 infection [74,75]. LL-37 at a concentration of 20 μg/mL can drastically reduce HSV-1 replication in Medical Research Council (MRC)-5 cells, and does not depend on the timing of LL-37 treatment (before, simultaneously, or after cells are challenged with HSV-1) [76]. In contrast, a study by Lee and coauthors has shown that LL-37 was unable to clear HSV spreading from already infected human corneal epithelial cells, and that it failed to control intracellular virus transmission, suggesting that LL-37 cannot be used as a therapeutic agent, but rather should be used as a prophylactic agent for the treatment of HSV-1 (Table 2) [77]. Liposomal LL-37 treatment in HSV-1-infected HaCaT (Human adult skin keratinocytes) cells showed broader antiviral activity at >25 μM concentrations. Similarly, liposomal LL-37 at a concentration of 20 μM or higher completely protects HSV-1-infected, immortalized keratinocytes (Ker-CT), with no detectable cell cytotoxicity. Nano-sized liposomal LL-37 was found to be significantly less toxic, capable of sustaining a long shelf-life (over 1 year at 4 °C), and rapidly taken up by the HaCaT cell line (50% achieved at 6 h after treatment) compared to free LL-37 peptides [78]. Therefore, liposomal-LL-37 can be used as a potential therapeutic agent for the prevention of HSV-1 infection; however, the mechanism behind this protection remains unknown [78].

Despite several beneficial effects of LL-37, documented through in vitro experiments, it has some detrimental effects on HSV-2-enhanced HIV transmission. HSV-2 augments the production of several AMPs, including LL-37 in normal human epithelial cells. Prior stimulation with LL-37 increases HIV susceptibility in monocyte-derived Langerhans cells by upregulating CD4 and CCR5 cell surface expression (Table 2). This study provides evidence on why HSV-2 infection enhances sexual transmission of HIV, and that it is probably due to the upregulation of LL-37 expression by epithelial cells [23]. In contrast, pretreatment of LL-37 in monocyte-derived DCs significantly upregulates CD86 and CCR7 expression, and decreases HIV infection in a dose-dependent manner [23], which suggests that the effect of LL-37 in regulating infection and virus transmission is cell-specific and receptor-dependent (Figure 1).

## 4. Effect of LL-37 on RNA Viruses

### 4.1. SARS-CoV-2 

With the novel beta coronavirus SARS-CoV-2 (severe acute respiratory syndrome-coronavirus-2), enveloped, single-stranded, positive-sense RNA virus leads to pandemic acute respiratory disease, pneumonia, and even death recently [79,80,81]. Evidence-based research findings have demonstrated an inverse correlation between the low level of serum 25-hydroxyvitamin D and the high risk of COVID-19 disease incidence or prevalence [82]. Vitamin D upregulates several antimicrobial agents, including LL-37, which can directly or indirectly act on SARS-CoV-2 and prevent its replication, increase anti-inflammatory cytokine production and decrease proinflammatory immune responses. Vitamin D deficiency in the African-American population has also been associated with a high risk of severe disease and SARS-CoV-2-induced mortality [83]. Mesenchymal stem cells (MSCs) and its derivatives are being tested in more than 20 clinical trials for the cure of SARS-CoV-2 infection [84]. MSCs produce several soluble factors, including the antimicrobial peptide LL-37, which can bind to the virus and disable its replication, whereas inactivated LL-37s are unable to bind the virus and fail to inhibit its replication [84,85]. In a recent study, 11 SARS-CoV-2 infected patients (six patients were RNA-positive and five were RNA-negative) were orally treated with recombinant *Lactococcus lactis* containing LL-37 peptide (1 × 10^9^ CFU/capsule, three capsules/time, three times a day for three weeks). Improvement of gastrointestinal, systemic, and respiratory symptoms in all LL-37-treated patients indicate the usage of LL-37 to be a safe and effective therapeutic approach for the cure of SARS-CoV-2 infection [86]. However, further randomized clinical studies are needed to prove this hypothesis and its impact on SARS-CoV-2 pathogenesis and prevention. The antiviral mechanism of LL-37 against SARS-CoV-2 also needs to be explored. 

### 4.2. Dengue Virus (DENV)

Dengue fever is a mosquito-borne tropical disease caused by an enveloped-RNA dengue virus (DENV) in the family *Flaviviridae*. Dengue fever is endemic in more than 100 countries, with major cases reported from southeast Asia, western specific regions, and the Americas [87]. The initial study with in vitro DENV-2 infection in THP-1 cell lines showed increased LL-37 mRNA expression at 6–48 h after infection, and protein increased up to 72 h after infection. Similarly, human neutrophils infected with DENV-2 showed an increase of LL-37 mRNA expression 2–5 h after infection [88]. Preincubation of DENV-2 with 10–15 μM of LL-37 before infection in in vitro Vero E6 cell lines showed a significant reduction in the viral RNA level, as well as NS1 antigens in the culture supernatant compared to the virus control (Table 2) [89]. However, no effect on percentage of infection and viral RNA level was detected in the cultures where LL-37 was added 24 h after viral infection or during the pretreatment of cells before infection, in comparison to the virus control. Molecular docking studies suggest that LL-37 inhibits DENV-2 infection by binding to the E protein dimers of the virus, which will eventually prevent cell–virus interaction and infection [89]. Pretreatment with 10–30 μg/mL of LL-37 has substantially reduced DENV-2 replication, and 30 μg/mL of LL-37 have completely inhibited virus multiplication in the HaCaT skin keratinocyte cell lines. DENV-2 infection in HaCaT cells upregulates IFNβ, IFNλ, IL-6, and IL-8, as well as LL-37 AMPs (Figure 1). The in vitro studies suggest that IFN I, IFN III, and LL-37 are important innate immune responses that may contribute to the protection in the skin during DENV infection [90].

### 4.3. Hepatitis C Virus (HCV)

HCV, an enveloped, single-stranded, positive-sense RNA virus under the family *Flaviviridae*, is one of the major causes of chronic liver disease, and has affected 71 million people around the world [91]. The physiological LL-37 in plasma ranges from 1.2 to 1.4 μg/mL in healthy individuals [92]. The pretreatment of Huh-7 cell lines with LL-37 at the concentration of 2–5 μg/mL attenuates HCV infection approximately two-fold, and at the concentration of 10–20 μg/mL reduced HCV infection further by 10-fold [93]. In contrast, an in vitro study using the BE-KO (Apolipoprotein B and Apolipoprotein E double-gene-knockout) cell line showed that exogenous expression of CAMP and LL-37 had no effect on intracellular HCV RNA levels; rather, it significantly increased the extracellular and intracellular HCV infection titers. Suggesting that a dose-dependent future study is needed to understand the antiviral role of LL-37 in HCV replication [94]. Chun and their teams [95] also observed increased mRNA levels of cathelicidin in HCV-positive patients with psoriasis, compared to HCV-negative patients with psoriasis. HCV infection upregulates cathelicidin, TLR9, and IFN-γ expression, which increases the susceptibility to develop psoriasis [95]. A significant increase in plasma LL-37 concentration has also been detected in HCV- and hepatitis B virus-infected patients compared to uninfected controls [96]. However, the increase in LL-37 levels in plasma does not correlate with the serum vitamin D level [96]. On the contrary, synthetic calcitriol derived calcipotriol had been shown to inhibit HCV replication by inducing vitamin D receptor target genes like cathelicidin and hepcidin. However, the underlying mechanism of inhibiting HCV replication by calcipotriol remains unknown [97].

### 4.4. Ebola Virus (EV)

Ebola virus (EV), a single-stranded, negative-sense RNA virus from the family *Filoviridae* has caused a major health emergency in the Democratic Republic of the Congo, with more than 3453 cases of infection and 2264 deaths recently [98]. Several preventive vaccines like rVSV-ZEBOV against EV are either approved or in different phases of clinical trials. The role of LL-37 and engineered LL-37 variants (GI-20 and 17BI) in inhibiting the infection of recombinant VSV–Ebola–GP–GFP (Glycoprotein-Green Fluorescence Protein) and wildtype EV has also been documented recently [99]. These LL-37 agents target EV at the endosomal cell-entry step by impairing cathepsin B-mediated processing of EV glycoprotein in the HeLa (Henrietta Lacks) cell line (Table 2). These LL-37s were able to inhibit EV cell entry, but they were not able to control virus replication. More importantly, two engineered cathelicidins derived from the antimicrobial peptides containing D-amino acids are resistant to intracellular enzymatic digestion, and are more potent than the L-form AMPs [99]. These promising results suggest further research to explore their role in preventing EV infection in animal models, as well as their possible use in combination with antiviral drugs, including the neutralizing antibodies [100,101].

### 4.5. Zika Virus (ZiKV)

Zika virus, an enveloped neurotropic flavivirus, which is mainly transmitted by Aedes mosquitoes [102], was declared by the World Health Organization as a public health emergency of international concern in 2016 [103]. Although ZiKV is asymptomatic in healthy adults, it mostly causes congenital abnormalities in the infants of infected mothers. A high risk of sporadic outbreak continues despite the substantial reduction in ZiKV infection since 2016. Nine AMPs, including LL-37, LL-37-derived peptides (GI-20, GI-20D-form, GF-17, 17BIPHE2, Merecidin, and RI-10), bovine cathelicidin BMAP-27-derived BMAP-18, and DASamP2 were tested in Vero cells to determine their anti-ZiKV effects in vitro. All nine peptides showed a dose-dependent decrease in ZiKV RNA levels compared to untreated controls, where Vero cells were pretreated with AMPs. BMAP-18, GF-17, and RI-10 were also able to inhibit zika infection in primary human fetal astrocytes cultures (Table 2). GF-17 and BMAP-18 do not affect viral attachments, but they interfere with the ZiKV entry at 2 h post-infection by significantly increasing and decreasing IFN-α and IFN-β1 expression, respectively, in a dose-dependent manner. Moreover, GF-17 was found to inactivate ZiKV by direct interaction in a time- and dose-dependent manner. Direct incubation of GF-17 at 10 μM concentration with ZiKV for 4 h caused a reduction in the majority of the virions compared with those treated with GF-17 at its lower dose and shorter incubation time. Therefore, LL-37 may be used as a potential therapeutic approach for future outbreaks where LL-37 will reduce the virus entry to cells by inactivating virus particles [104].

### 4.6. Human Rhinovirus (HRV)

Human rhinoviruses (HRVs), non-enveloped, positive-sense, single-stranded RNA viruses from *Picornaviridae*, are responsible for the “common cold” and are a major cause of mortality and morbidity worldwide [105]. HRVs enter the epithelial cells via endocytosis after binding with either intercellular adhesion molecule-1 (ICAM-1), low-density lipoprotein receptors, or the cadherin-related family member 3 [106,107]. Vitamin D (both 25(OH)D and 1,25(OH)_2_D)-induced resistance to HRV infection is associated with the induction of the CAMP expression. CAMP is a vitamin D-inducible gene that encodes the hCAP-18 protein from which LL-37 is derived [108,109]. LL-37 has direct antiviral effects against HRV in an in vitro model where the virus had been incubated with LL-37 prior to infecting alveolar epithelial cells. LL-37 treatment significantly reduces cell metabolic activity at lower concentrations of LL-37 (≤30 μg/mL) in infected cells compared to healthy cells, without enhancing cell apoptosis or necrosis [110]. The pretreatment of LL-37 to lung epithelial cells effectively reduces HRV replication and the release of infected virion in the cell culture supernatant (Table 2). However, the conversion of all positively-charged arginines to citrullines (citrullination), which is regulated by the level of peptidyl arginine deiminase (PAD) enzyme, resulted in the complete reduction of LL-37 antiviral activity against HRV replication [111]. Therefore, targeting to reduce the PAD enzyme may help to maintain LL-37 activity against HRV infection. 

Serum LL-37 levels were inversely correlated with HRV viral load in broncho-alveolar lavage of cystic fibrosis patients [112]. In contrast, a multicenter study with cohort of >100 infants showed that infants with highest quartile of serum LL-37 were less likely to have respiratory syncytial virus (RSV) bronchiolitis, but more likely to have HRV bronchiolitis [113]. 

### 4.7. Respiratory Syncytial Virus (RSV)

Respiratory syncytial viruses (RSV), single-stranded, negative-sense RNA viruses under the family *Paramyxoviridae*, are a leading cause of severe lower respiratory disease in infants and young children, with up to 149,400 deaths in children <5 years of age annually [114]. As of today, there is no vaccine available that can prevent this infection. LL-37 directly binds to RSV and causes a significantly low overall level of colocalization of the F- and N-proteins (representing partial or complete viral particle disruption) than controls in in vitro experiments [115]. In vitro administration of LL-37 in Hep-2 cells also significantly lowers the production of type I and type III IFNs, suggesting that the action of LL-37 is mediated by direct effect rather than by regulating the IFN responses. LL-37 has been reported to inhibit RSV replication by damaging the integrity of the RSV envelope and preventing cellular binding (Table 2). LL-37 has also been shown to have an antiviral effect against RSV infection in in vivo murine models by lowering IFN-β production, which again emphasizes that antiviral effects are not mediated through the modulation of IFN pathways [115,116]. 

### 4.8. Venezuelan Equine Encephalitis Virus (VEEV)

Venezuelan equine encephalitis virus, an enveloped, positive-sense, single-stranded RNA virus under the family *Togaviridae*, is a mosquito-borne viral agent that causes diseases of variable severity, ranging from mild febrile illness to life-threatening encephalitis [117]. LL-37 has been demonstrated to prevent virus replication by inhibiting the virus entry in an in vitro cell culture experiment (Table 2). LL-37 was found to modulate type I IFN expression, where the IFN-β1 expression increase was detected in infected cells [118]. This study provides a novel antiviral role for LL-37 in the treatment of early stage of VEEV infection. 

### 4.9. Human Immunodeficiency Virus (HIV)

Human immunodeficiency virus (HIV)-1 and HIV-2, an enveloped, single-stranded, positive-sense RNA virus from the genus Lentivirus causes AIDS. An estimated 1.7 million people became recently infected with HIV, and 770,000 people died from AIDS-related illness in the year 2018. The discovery of antiretroviral therapy (ART) has drastically improved HIV patient mortality, yet this success is accompanied by a dramatically increased incidence of cardiovascular diseases, accelerated aging, and liver-related comorbidities. LL-37 and its fragments LL13-37 and LL17-32 can directly bind to HIV-1 reverse transcriptase to block its activity in a dose-dependent manner in in vitro experiments (Table 2) [119]. A vitamin D and phenylbutyrate supplement study in ART-naïve patients had significantly increased 25(OH)D levels in plasma after 16 weeks of treatment; however, there were no changes in the serum LL-37 level, as well as no improvement in gut-derived immune activation markers [120]. Depot medroxyprogesterone acetate (DMPA), a popular contraceptive used in sub-Saharan Africa was found to be associated with increased HIV acquisition, suggesting that a randomized controlled trial is needed to understand the link between the use of DMPA and HIV risk [121]. In contrast, DMPA administration significantly increased serum LL-37 concentration (0.81 vs. 0.40 log_10_ ng/mL; *p* = 0.027), suggesting that this treatment may actually provide increased antiviral activity in the female genital tract and may reduce HIV acquisition, which needs additional future studies [122]. 

Antimicrobial peptide LL-37 produced by epithelial cells also accelerates CD4 and CCR5 expression in Langerhans cells and accelerated HIV infection in an ex vivo explant culture (Table 2, Figure 1) [23]. Higher levels of cathelicidin LL-37 in cervicovaginal secretions (CVSs) have been positively associated with increased HIV acquisition in a subgroup of female sex workers where the sex workers had increased rates of genital infections, which might have accelerated HIV susceptibility (Table 2) [123]. CVSs collected from HIV-exposed seronegative, HIV-seropositive, and low-risk controls were found to have HIV neutralizing activity, and a selective depletion of LL-37 peptide demonstrated significantly reduced functional activity. Similarly, human CVS lacking intrinsic HIV neutralizing activity can be restored by the addition of recombinant LL-37 peptides in in vitro experiments [124].

In ART-untreated, HIV-infected patients, LL-37 levels were consistently low for any given vitamin D levels compared to healthy controls. Again, ART-untreated HIV+ patients have significantly lower plasma LL-37 levels compared to the ART-treated HIV positive patients, suggesting that ART may have reduced HIV replication to rescue plasma LL-37 levels [125,126]. The level of plasma LL-37 was found to be positively correlated with the proinflammatory cytokine IL-6 [125]. All of these studies have suggested that a balanced level of LL-37 might be beneficial for the control of HIV acquisition: the increased level of LL-37 may upregulate the receptor and the coreceptors of the target cells and influence HIV replication and infection (Table 2). However, a detailed in vivo animal experiment is necessary to determine the beneficial effect of LL-37 in HIV infection.

## 5. Conclusions

LL-37, like other AMPs, exhibits antimicrobial activities against a broad spectrum of microbes, including bacteria, enveloped viruses, and fungi. Due to their antimicrobial properties, AMPs have been a promising target in infections treatment as alternatives to systemic antibiotics [66]. However, the administration of manufactured peptides may induce microbial resistance to innate human defenses against microbial invasion, as demonstrated for various antibiotics with cathelicidin-like properties [67,68].

Nowadays, LL-37, in addition to its antimicrobial properties, can influence and modulate, both directly and indirectly, the activity of various cell populations involved in inflammatory processes, and intensify the course of inflammation by attracting neutrophils, monocytes, macrophages, eosinophils, and mast cells to the pathogen entry site. Importantly, epidermal keratinocytes and neutrophils are a rich source of LL-37, and may have a pivotal role in triggering the early inflammatory events of autoimmune diseases. Given the emerging pathogenic role of LL-37 in autoimmune diseases, this molecule could be a potential target for immune modulation as well. Neutralization of TLR4 signaling holds promise for diseases, such as colitis, chronic pain, and sepsis [69]. Given the effects of LL-37 on this receptor, it may have therapeutic potential in diseases influenced by TLR4 signaling. For diseases where LL-37 may activate TLR7 and TLR9 signaling, such as psoriasis and SLE, an antagonist drug of LL-37 may provide a novel therapeutic strategy. Because LL-37 is able to participate in immune complex formation [16], the neutralization of LL-37 by antibodies should be properly evaluated. 

The antiviral activity of LL-37 works in different ways, including virus–cell interaction, direct inactivation of virus particles, modulating type I and III IFN production, upregulating ISGs, preventing virus replication, disrupting virus particles and protein processing, and possibly facilitating the cellular communications that regulate adaptive immune responses. On the other side, the increased production or administration of LL-37 may accelerate the production of proinflammatory cytokines and increase the expression of some key cell receptors that augment increased viral replication and infection. A detailed understanding of the mode of action of LL-37 using animal models is crucial. The majority of LL-37 studies have been performed in in vitro cell culture models, suggesting that LL-37 may be more beneficial as a prophylactic drug rather than as a therapeutic agent, which also needs future studies. Targeting the antiviral and immunomodulatory functions of LL-37 opens a new approach to limit virus dissemination and the progression of disease.

Overall, although cathelicidins have been discovered nearly 30 years ago, the elucidation of new properties and functions in recent years continues to provide more insight into the physiological and pathogenic roles and potential applications of this immunomodulatory and anti-microbial peptide.

## Figures and Tables

**Table 1:** Biological effects of LL-37 in psoriasis and lupus erythematosus.

LL-37-Mediated Pathogenic Events	Psoriasis	Lupus Erythematosus
LL-37 induces the release of inflammatory mediators by keratinocytes LL-37 induces keratinocyte migration and proliferation	+ +	+
LL-37 reduces keratinocytes apoptosis	+	
LL-37/DNA complexes as autoantigens	+	+
LL-37/DNA complexes stimulate pDCs to high IFN-α production	+	+
LL-37/RNA complexes stimulate mDCs to high TNF-α and IL-6	+	
Presence of LL-37-specific, IFN-γ positive CD4^+^ and CD8^+^ T cells	+	
LL-37-specific T cells promote anti-LL-37 antibody production by B cells		+

**Table 2:** LL-37-induced antiviral and inflammatory responses.

Category	Functions	Viruses
Favorable	Prevents virus entry shown by preincubation	Venezuelan equine encephalitis virus (VEEV), respiratory syncytial virus (RSV), dengue virus (DENV), Zika virus (ZiKV), Ebola virus (EV), hepatitis C virus (HCV) (?)
	Type I and/or III IFN modulation	VEEV, ZiKV
	Viral particle disruption and protein processing	RSV, EV
	Direct antiviral effect	Human rhinovirus (HRV), ZiKV, human immunodeficiency virus (HIV), VV, RSV
	Suppresses reverse transcriptase enzyme activity	HIV
	Upregulation of interferon-stimulating genes (ISGs)	Herpes simplex virus 1 (HSV-1)
	Prevents viral replication	HSV-1 (?)
Unfavorable	HSV-2-induced LL-37 upregulation increases virus susceptibility	HIV
	LL-37 upregulated CD4 and CCR5 expression in Langerhans cells increases virus susceptibility	HIV
